# Hepatic portal venous gas in the case of superior mesenteric artery thrombosis in a young adult‐case report

**DOI:** 10.1002/ccr3.6989

**Published:** 2023-02-24

**Authors:** Prakash Dhakal, Suraj Sharma, Abhishek Sharma, Shailendra Pandey, Sajiva Aryal, Seema Bhnadari

**Affiliations:** ^1^ Department of Radiology, National Academy of Medical Sciences Bir Hospital Kathmandu Nepal; ^2^ Tribhuvan University Institute of Medicine Kathmandu Nepal; ^3^ Kathmandu University Dhulikhel Nepal; ^4^ Tilganga Institute of ophthalmology Kathmandu Nepal

**Keywords:** hepatic portal venous gas, intestinal perforation, Pneumatosis intestinalis, superior mesenteric artery (SMA) thrombosis

## Abstract

Hepatic portal venous gas is diagnosed via computed tomography due to unusual imaging features. HPVG when linked with pneumatosis intestinalis has a high mortality rate and required urgent intervention. We present a case of a 26‐year‐old young adult with superior mesenteric artery thrombosis who presented with severe abdominal pain. On imaging, HPVG and pneumatosis intestinalis were seen owing to the urgent intervention of the patient. The reliable interpretation of the imaging findings along with quick intervention led to a favorable outcome in our case. Herein, we present a thorough review of the imaging findings of HPVG to make a reliable diagnosis when presented with such a case.

## Graphical abstract

HVPG is a rare condition that might be difficult to cure. Is significantly more difficult to treat and has a greater death rate when it is accompanied by intestinal necrosis. In our case, superior mesenteric artery thrombosis led to bowel ischemia.

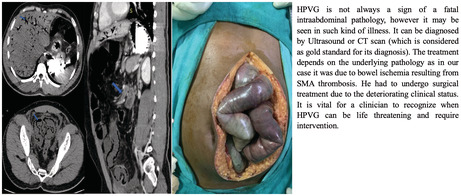

## 1 INTRODUCTION

Gas in the portal venous system and its branches is a pathological disease known as hepatic portal venous gas (HPVG). The clinical symptoms of HPVG ranges from diseases that are benign to life‐threatening.^1^ This unusual imaging feature serves as a diagnostic indicator of serious abdominal illness.^2^

While the presence of either HPVG or Pneumatosis Intestinalis (PI) on a CT scan alone does not always indicate that there has been a bowel infarction, the presence of both of these illnesses on a CT scan is strongly linked to this condition, particularly if there is band‐like pneumatosis. It typically denotes irreparable damage and transmural necrosis.^3^ A reported 75% mortality rate for HPVG linked with PI indicates that it is a life‐threatening condition.^2^

## 2 CASE REPORT

A 26‐year‐old male patient presented to our emergency department with abdominal pain. The pain was gradual in onset, intermittent in nature for a month, which suddenly increased in severity for a day. The pain was not referred to other parts of body. He also had nausea and vomiting associated with it. However, he did not complain of fever. His examination revealed blood pressure to be 130/70 mm hg, heart rate of 100 bpm, respiratory rate of 22 per minute, temperature of 98 °F, and SpO2 93%. Abdominal examination revealed marked abdominal tenderness diffusely. In addition, he had guarding and rigidity present.

Laboratory examination showed the following:

**Table:** 

Total white blood cell count	9.4 × 10^9^/L
Neutrophils	68.1%
Hemoglobin	13gm%
Platelet	190,000/cumm
Arterial blood gas analysis	
pH	7.4
PaO_2_	98
PCO_2_	42
HCO_3_^−^	23

Following the laboratory examination, a CT scan of the abdomen was done.

## 3 IMAGING FINDING

### 3.1 CT finding of the patient

**FIGURE 1:**
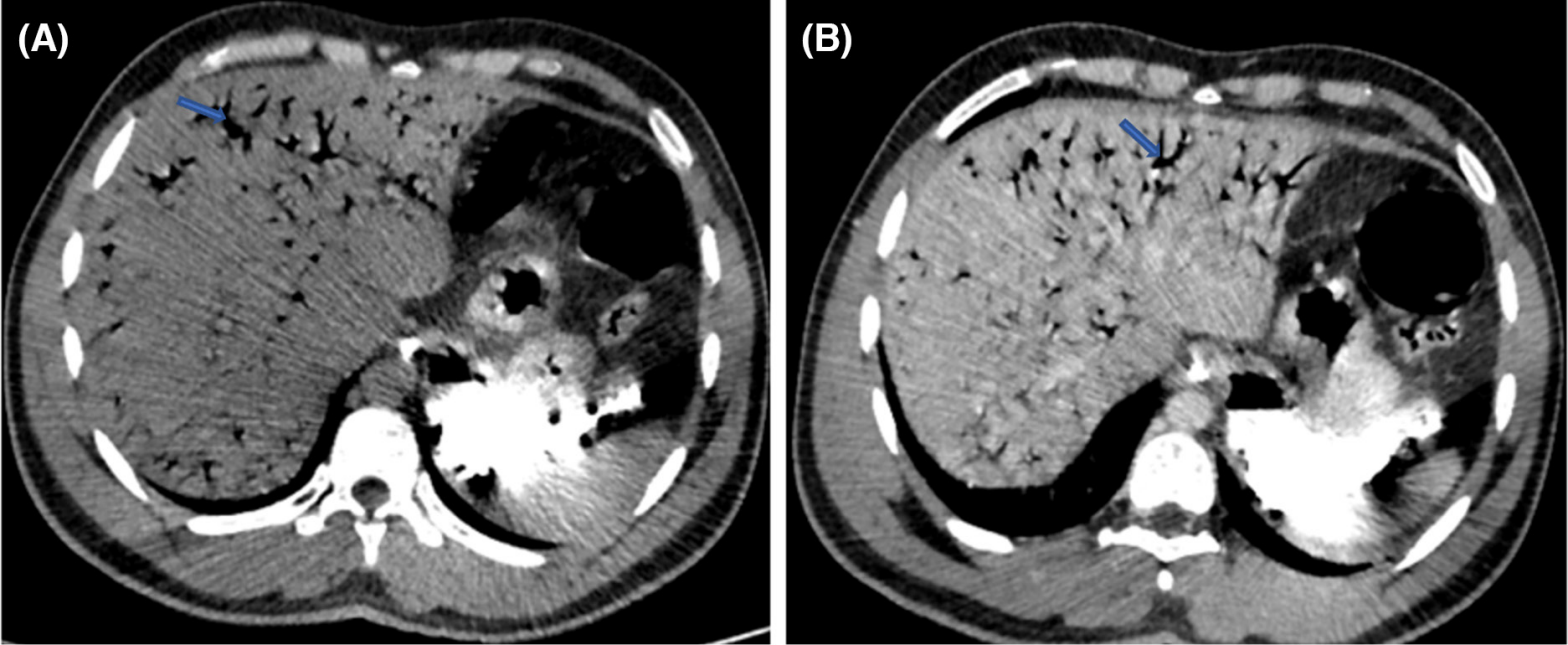
(A): axial noncontrast; (B): axial postcontrast CT images demonstrating linear branching hypoattenuating (HU‐998) areas (arrow) in both lobes of liver extending up to the periphery and in continuation with portal venous branches.

**FIGURE 2:**
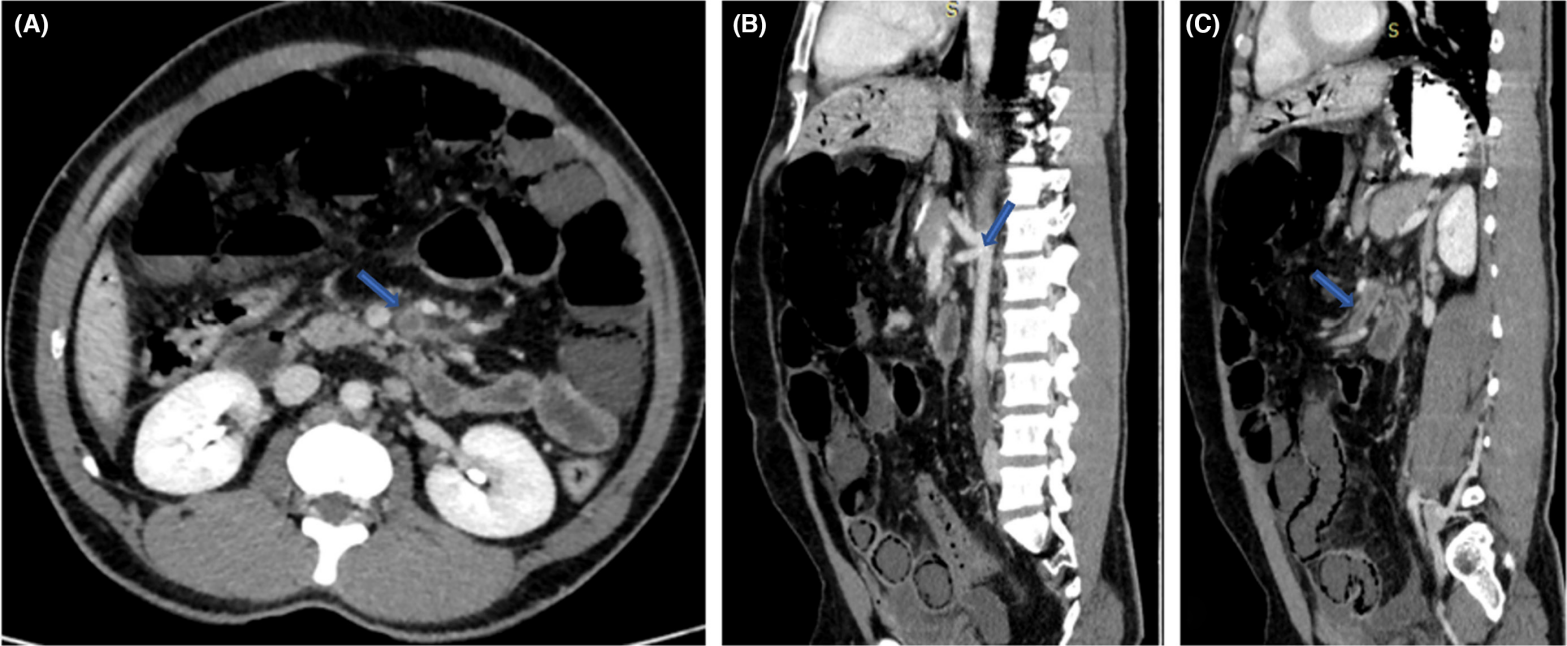
(A): Postcontrast axial CT image demonstrating filling defect in superior mesenteric artery (arrow). (B): Postcontrast sagittal CT image demonstrating celiac trunk and superior mesenteric artery with contrast opacification in their proximal aspect (arrow). (C): Postcontrast sagittal CT image demonstrating nonenhancing filling defect in superior mesenteric artery starting from approximately 2 cm distal to its origin (arrow). Figure 2 (B) and (C) also demonstrates air attenuating areas within the bowel wall (pneumatosis intestinalis) and minimal free fluid in pelvic peritoneal cavity.

**FIGURE 3:**
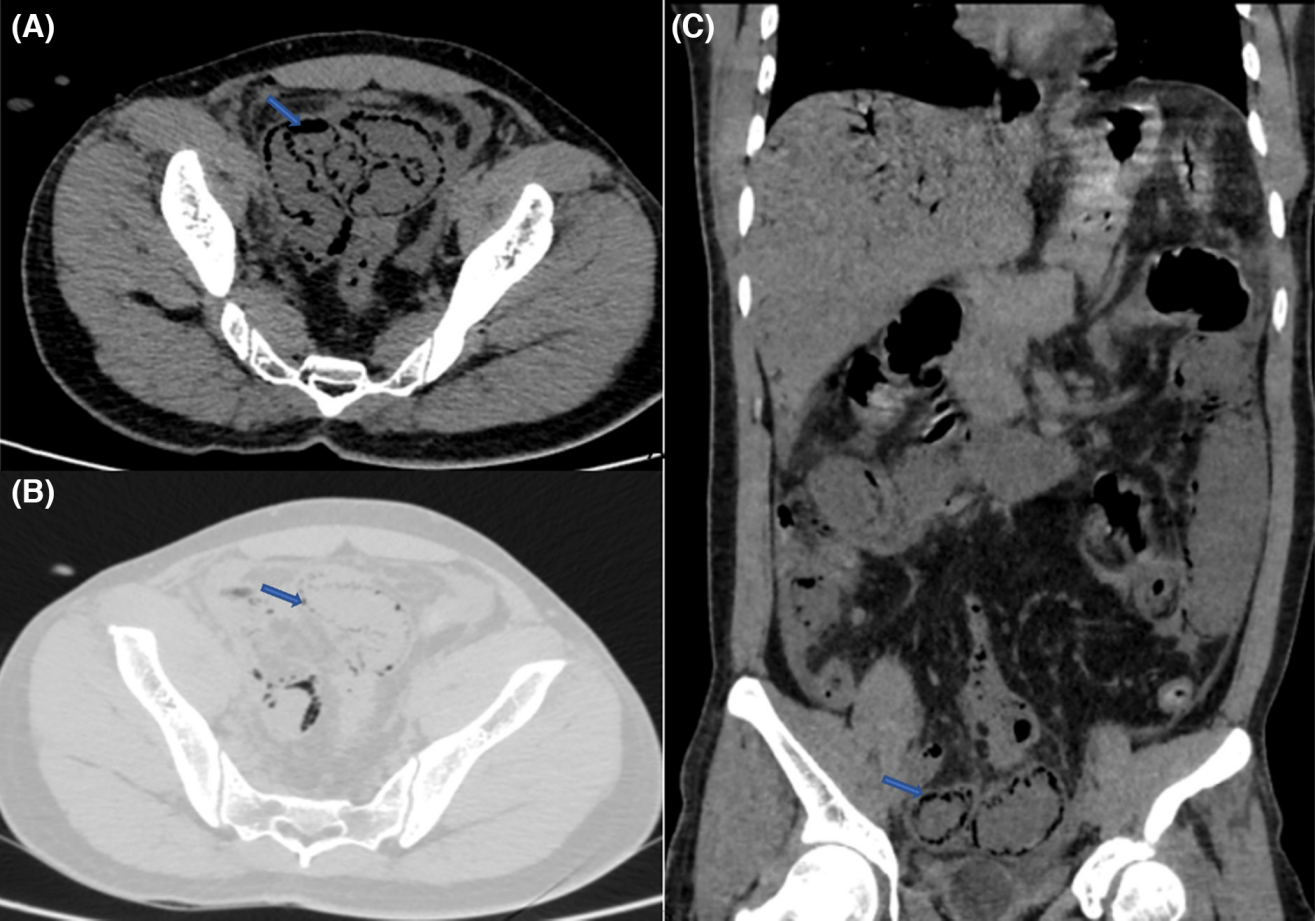
(A): axial noncontrast (soft tissue window), (B): axial noncontrast (lung window), and (C): coronal noncontrast CT images showing thinning of bowel walls in the pelvic region (probably small bowel loops) with multiple round cystic air attenuating areas within the bowel wall (arrows) demonstrating pneumatosis intestinalis.

Noncontrast and contrast CT of abdomen and pelvis were done, which demonstrate linear branching air attenuating areas (HU‐998) in both lobes of liver involving periphery and in the region of portal venous branches, these features are suggestive of portal venous gas (Figure 1). Postcontrast study shows near total non‐enhancing filling defect in superior mesenteric artery starting from approximately 2 cm distal to its origin (Figure 2). There was thinning of bowel loops in pelvic region (probably small bowel loops) with hypoenhancing walls and multiple round cystic air attenuating areas within the bowel walls demonstrating pneumatosis intestinalis (Figure 3). Minimal free fluid is noted in pelvic peritoneal cavity.

## 4 IMAGING DIAGNOSIS

Superior mesenteric artery thrombosis leads to the bowel ischemia along with pneumatosis intestinalis and hepatic portal venous gas.

## 5 DISCUSSION

Hepatic portal venous gas (HPVG) is a rarely described form of pneumatosis and refers to gas within the portal vein.^4^ Radiologically, linear branched radiolucencies that reach the liver's edge within 2 cm are referred to as HPVG.^2^ The first case of HPVG was described in 1955 by Wolf and Evans.^5^

Mucosal damage, bowel distension, and sepsis caused by gas‐forming bacteria are the three possible mechanisms of HPVG. A necrotic bowel was present in more than two‐thirds of patients with hepatic portal venous gas. Pneumatosis intestinalis (PI), subserosal and submucosal gas‐filled cysts in the digestive tract, is frequently accompanied by hepatic portal venous gas.^2^, ^6^, ^7^ Numerous fatal and non‐fatal diseases and disorders, including intestinal necrosis, total or partial bowel obstruction, intraperitoneal abscess, ulcerative colitis, gastric ulcer, Crohn disease, trauma, endoscopic procedure complications, and diverticulitis, are associated with HPVG. The main cause of HPVG is ischemic bowel with subsequent intestinal necrosis.^2^

HVPG is a rare occurrence, and it is challenging to treat.^6^ It is even more challenging and has higher mortality when it is associated with bowel necrosis as reported by Kinoshita et al.^5^ In our case, HPVG along with Pneumatosis Intestinalis manifested as a result of bowel ischemia due to superior mesenteric artery (SMA) thrombosis, which later caused the necrosis.

HVPG is diagnosed radiologically using ultrasound or computed tomography (CT).^5^ However, CT has higher sensitivity for its diagnosis among all and is used as gold standard for its diagnosis.^2^, ^5^ Ultrasound shows either echogenic particles flowing within the portal vein or poorly defined, echogenic patches within the hepatic parenchyma, mostly in nondependent part.^5^ It is possible to dynamically image the centrifugal flow of portal gas to the hepatic periphery using color Doppler flow imaging, differentiating it from biliary gas.^2^ In CT imaging, HPVG appears as branching lucencies that extend to within 2 cm of the liver capsule, primarily in the anterior‐superior portion of the left lobe. As opposed to biliary gas (pneumobilia), which is linked to air in the liver's center but does not reach as far toward the liver capsule as does HPVG (air in HPVG extends to a less than 2 cm from the liver capsule, whereas in pneumobilia it does not reach to that extent), pneumobilia is characterized by air within the liver's central region.^8^ HPVG was diagnosed using CT imaging in our case, which showed shows near total nonenhancing filling defect in superior mesenteric artery starting from approximately 2 cm distal to its origin (Figure 2). It was also associated with the finding of PI seen as was thinning of bowel loops in pelvic region (probably small bowel loops) with hypoenhancing walls and multiple round cystic air attenuating areas within the bowel walls (Figure 3).

**FIGURE 4:**
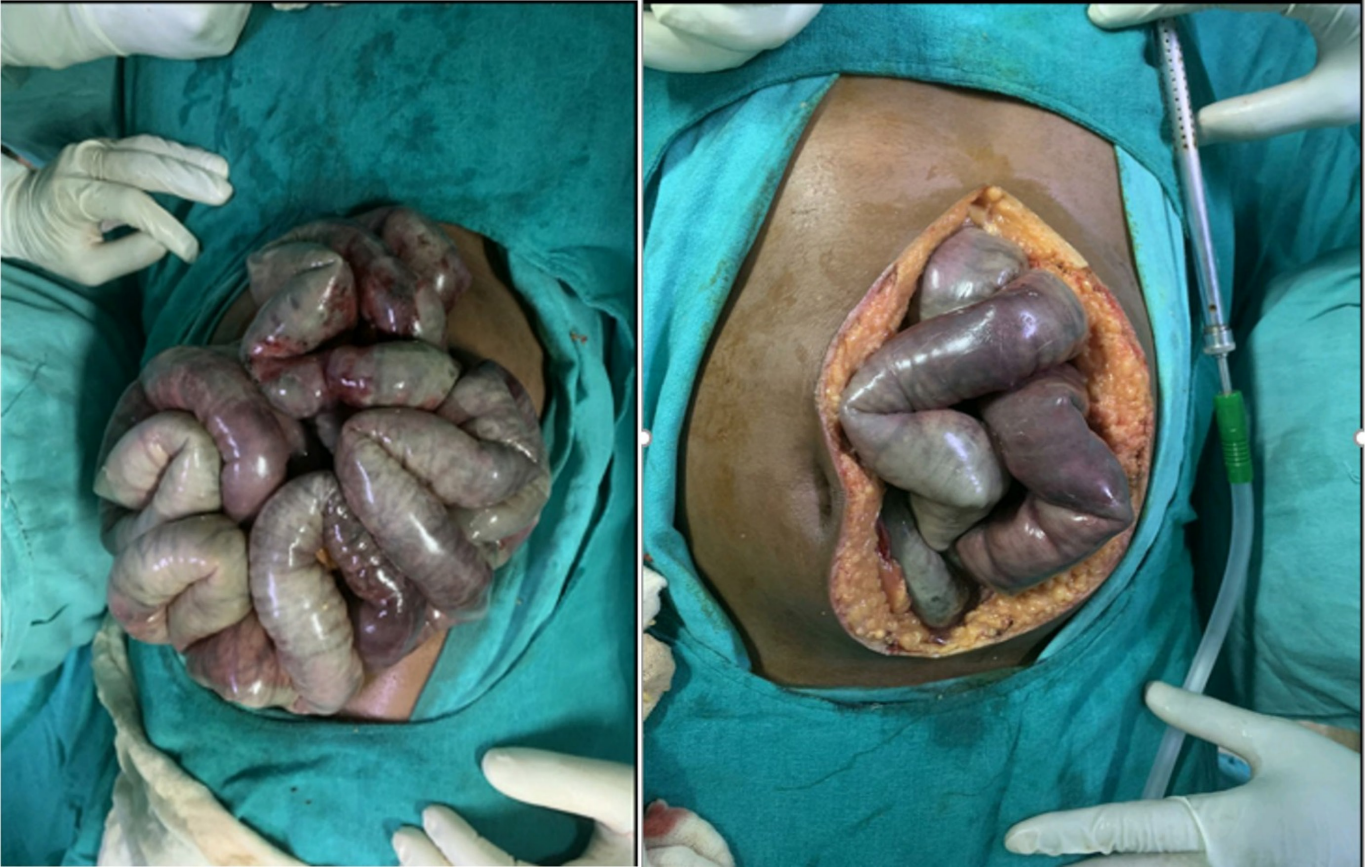
Image of the necrosed bowel revealed after surgery.

Treatment of HPVG mostly depends upon the underlying cause. It also depends on the presence or absence of peritonitis or intestinal perforation, as well as the patient's general condition, as they are the primary characteristics that direct clinicians in their therapeutic strategy.^9^ Our patient's condition was deteriorating. He was resuscitated with intravenous fluid and antibiotics. With the diagnosis of the bowel ischemia due to SMA thrombosis, he underwent emergency explorative laparotomy and resection and anastomosis was done (Figure 4). Fortunately, he became stable and improved after the surgery.

## 6 CONCLUSION

HPVG is not always a sign of a fatal intraabdominal pathology; however, it may be seen in such kind of illness. It can be diagnosed by Ultrasound or CT scan (which is considered as gold standard for its diagnosis). The treatment depends on the underlying pathology as in our case it was due to bowel ischemia resulting from SMA thrombosis. He had to undergo surgical treatment due to the deteriorating clinical status. It is vital for a clinician to recognize when HPVG can be life‐threatening and require intervention.

## AUTHOR CONTRIBUTIONS

**Prakash Dhakal:** Supervision. **Suraj Sharma:** Visualization. **Abhishek Sharma:** Writing – review and editing. **Shailendra Pandey:** Writing – review and editing. **Sajiva Aryal:** Writing – original draft. **Seema Bhnadari:** Writing – original draft.

## FUNDING INFORMATION

No funds were received from any institutions or any persons for this case report.

## CONFLICT OF INTEREST STATEMENT

There is no conflict of interest among authors.

## ETHICAL APPROVAL STATEMENT

Not applicable.

## INFORMED CONSENT

Written informed consent was obtained from the patient to publish the report in accordance with the journal's patient consent policy.

## Data Availability

The datasets used and/or analyzed during the current study are available from the corresponding author (Dr.Prakash Dhakal) upon reasonable request.
